# Influence of Tea, Coffee, and Turmeric Solutions on the Color Stability of Denture Base Acrylic Resins and Silicone Soft Lining Material: An In Vitro Study

**DOI:** 10.7759/cureus.89653

**Published:** 2025-08-08

**Authors:** Geethu R M, Anil Kumar S

**Affiliations:** 1 Prosthodontics, Government Dental College Thiruvananthapuram, Trivandrum, IND; 2 Prosthodontics, Kerala University of Health Sciences, Thrissur, IND

**Keywords:** cie lab colour system, colour stability, denture base acrylic resin, polymethyl methacrylate, soft liner

## Abstract

Background and objectives

With the continuous presence of microflora, saliva, and frequent intake of coloured food, the colour stability of any aesthetic material may become compromised. Hence, the present study was conducted to evaluate the influence of tea, coffee, and turmeric solutions on the colour stability of commercially available heat-cured and autopolymerizing denture base acrylic resins as well as a soft lining material.

Methods

Twenty-four rectangular samples measuring 20 mm × 15 mm × 2 mm were prepared for each type of test material. The samples were divided into four groups of six and immersed in different staining solutions, then stored in an incubator at 37°C for 30 days. Colorimetric measurements were taken on the 1st, 7th, and 30th days using an ultraviolet-visible-near infrared (UV-VIS-NIR) spectrophotometer and reported according to the Commission Internationale de L’Eclairage (CIE) Lab system. Data were statistically analyzed using ANOVA, followed by the Tukey honestly significant difference (HSD) test. Comparisons between time intervals were performed using the paired t-test.

Results

In the heat-cure acrylic group, significant colour changes were caused by tea and coffee solution after days 7 and 30 (P < 0.001*) and by turmeric solution at all three time intervals. In the autopolymerizing acrylic group, significant colour changes were observed in turmeric and coffee solutions after days 7 and 30 and in tea solution after day 1 (P < 0.001*). For Molloplast-B, significant colour changes were seen in tea after day 7 (P < 0.001*) and in coffee and turmeric solutions at all three time intervals.

Interpretation and conclusion

Within the limitations of this study, it was concluded that significant colour shifts occurred in all three materials over time. The staining becomes more intense with time, except for autopolymerizing acrylic in tea and heat-cure acrylic in coffee. All the mean colour shifts were clinically acceptable, except for heat-cure acrylic in tea after 30 days.

## Introduction

Restoration of a missing tooth with a dental prosthesis is the ideal treatment to recreate a natural smile in completely or partially edentulous patients. Denture base polymers have been effectively used over several years for the fabrication of complete or partial dentures [1]. Polymethyl methacrylate (PMMA) resin, first introduced by Dr Walter Wright in 1937, has reliably performed well since its launch for the construction of dentures due to its varied advantages, such as ease of workability, low cost, satisfactory physical and mechanical properties, biocompatibility, and pleasing appearance [2]. Ease of shade matching makes it the preferred material for denture prosthesis [3]. However, this material has certain inherent weaknesses such as reduced strength, low fracture resistance, lack of elasticity, poor abrasion resistance, porosity, and low colour stability [2].

Soft denture liners are widely used for providing comfort to denture wearers who cannot adapt to a conventional denture base [4]. Even though these materials have favourable initial properties, they have certain shortcomings like discolouration, long-term resiliency, abrasion resistance, bonding failure, fouling by *Candida albicans*, odour, and porosity [5].

Colour is one of the premium properties of an aesthetic restorative material. The success or failure of the material depends on the preservation of harmonious colour for its entire lifespan [6]. As specified by various global standards, colour changes are indicators of the ageing of denture base polymers [7]. Changes in the optical properties of a polymeric material after long-term use may be due to intrinsic and extrinsic factors. Intrinsic factors refer to changes in the resin matrix and internal resin discolouration, while thermal changes, stain accumulation, artificial dyes used in food, cleaning procedures, and patient handling are the extrinsic factors [8]. Colour stability of a denture base material may become compromised due to the constant presence of microflora, saliva, and frequent intake of coloured food (chromatogens) [9].

Instrumental determination of colour changes permits objective readings, thus eliminating the personal interpretation of visual comparisons [9]. The Munsell Colour System and Commission Internationale de L’Eclairage (CIE L*a*b) are used to assess chromatic variations [6]. The CIE L*a*b colour space is a uniform colour distribution in 3D space, based on the blending of red, blue, and green colours in certain percentages. The extent of total colour difference, ΔE*, is the algebraic distance between two points in the colour space [6]. Previous research reported that colour differences exceeding 1 ∆E unit are perceptible to approximately 50% of human observers, while differences greater than 2 ∆E units are detectable by nearly all observers. Moreover, an average colour difference below 3.7 ∆E units is considered an acceptable match in clinical settings [10].

Colour changes in the oral environment need to be further explored to predict which materials will provide the best clinical service in long-term use. Hence, the present study was done to determine the effect of tea, coffee, and turmeric solutions on the colour stability of heat-polymerized resin, chemically polymerized resin, and soft denture silicone reline material.

## Materials and methods

**Figure 1:**
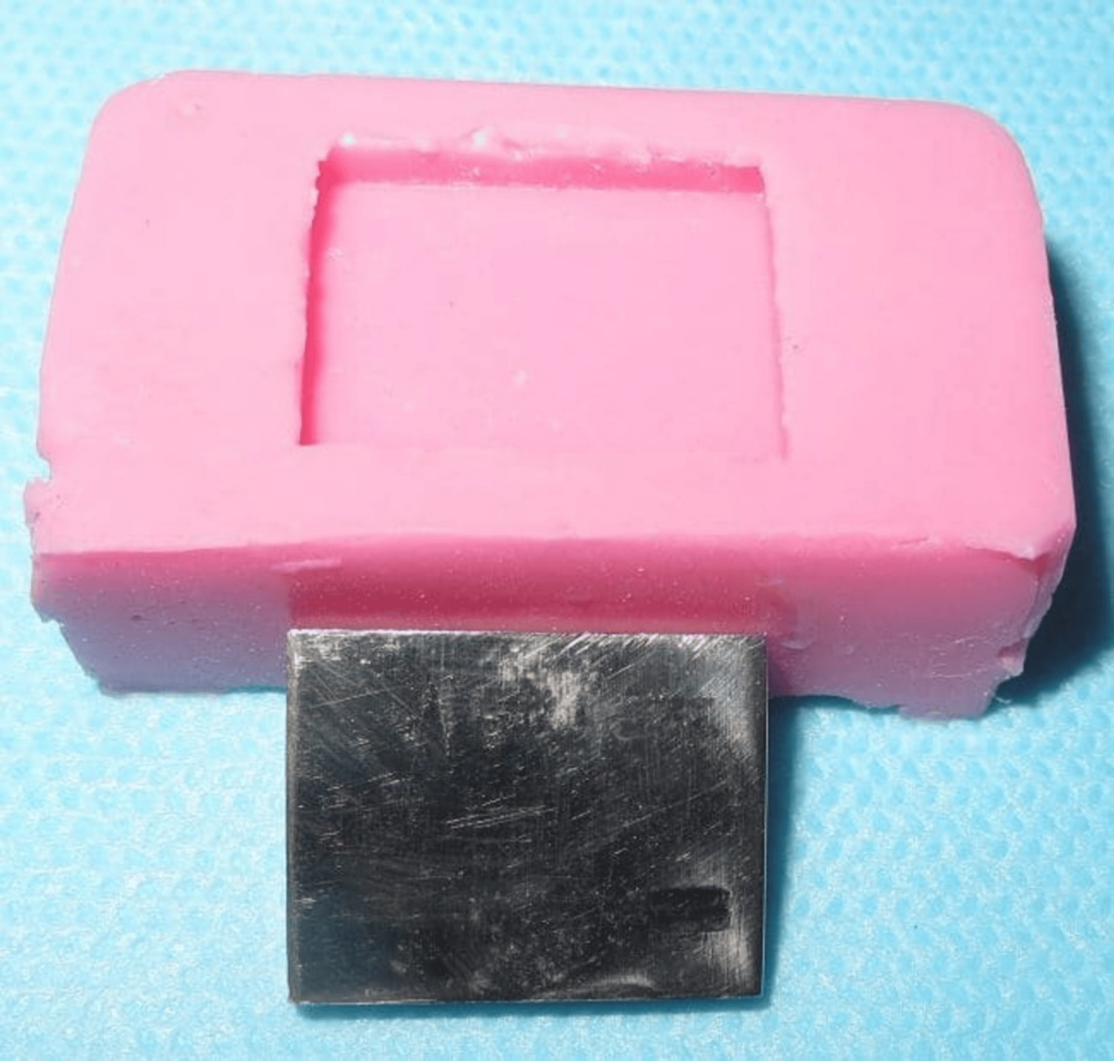
Stainless steel die and silicone mould of the die Master die of 20 mm length, 15 mm width, and 2 mm thickness prepared in stainless steel.

A master die measuring 20 mm in length, 15 mm in width, and 2 mm in thickness was prepared in stainless steel to fit the cuvette of the spectrophotometer (Figure 1).

Each group of 24 samples was fabricated using heat-cure polymethylmethacrylate resin (H), autopolymerizing polymethylmethacrylate resin (A), and Molloplast B (M) through the conventional flasking and compression moulding procedure, following the manufacturer’s recommendation (Table 1).

**Table 1:** Testing materials used for the study

Resin Used	Code	Number of samples prepared	Manufacturer
Heat-cure polymethyl methacrylate resin	H	24	Pyrax Polymers, Roorkee, India
Autopolymerizing polymethyl methacrylate resin	A	24	Pyrax Polymers, Roorkee, India
Soft lining material - Molloplast B	M	24	DETAX GmbH & Co. KG, Ettlingen, Germany

**Figure 2:**
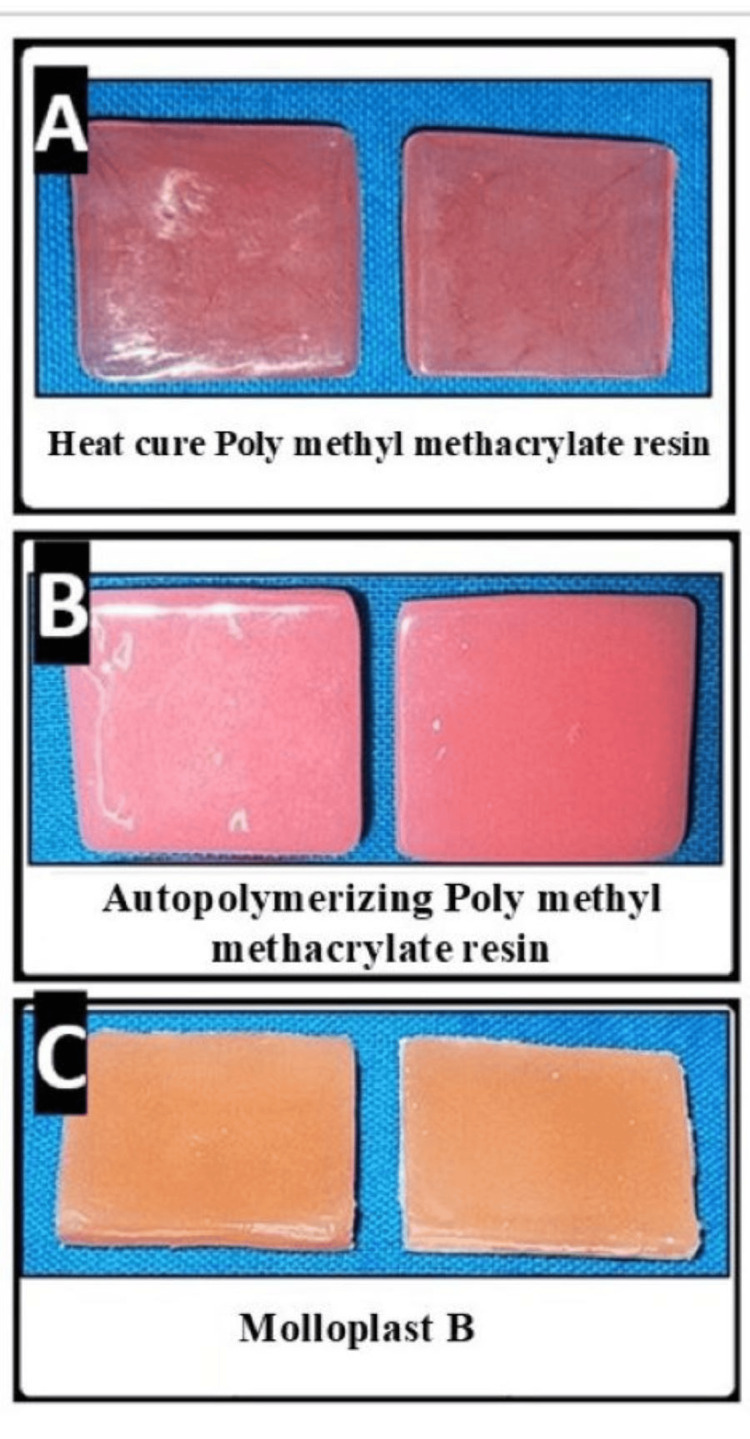
Testing samples prepared for the study (A) Heat-cure polymethyl methacrylate resin, (B) autopolymerizing polymethyl methacrylate resin, and (C) Molloplast B.

After polymerization, a smooth, glossy finish was achieved by finishing with sequential grits of silicon carbide paper (120 and 320) attached to a mandrel at a speed of 300 rpm for 1 minute for each sandpaper, followed by buff polishing with pumice slurry (Figure 2).

The prepared samples were immersed in distilled water for 24 hours to reduce the residual monomer content. Tea, coffee, and turmeric stains were prepared by dissolving 8 g of each colourant in 400 ml of boiling water. The solutions were allowed to cool for 10 minutes before filtration through a piece of gauze. Each staining agent was obtained by mixing 660 ml of artificial saliva and 330 ml of tea, coffee, and turmeric solutions, respectively (Table 2).

**Table 2:** Staining agents used for the study

Staining agent	Code	Manufacturer
Artificial saliva (Control)	AS	MP Sai Enterprises, Mumbai, India
Tea	T	Brooke Bond Red Label Hindustan Unilever Ltd., Mumbai, India
Coffee	Co	Nescafé Sunrise Premium, Nestle India Ltd., New Delhi, India
Turmeric	Tu	Everest Turmeric Powder, Everest Spices, Mumbai, India

**Figure 3:**
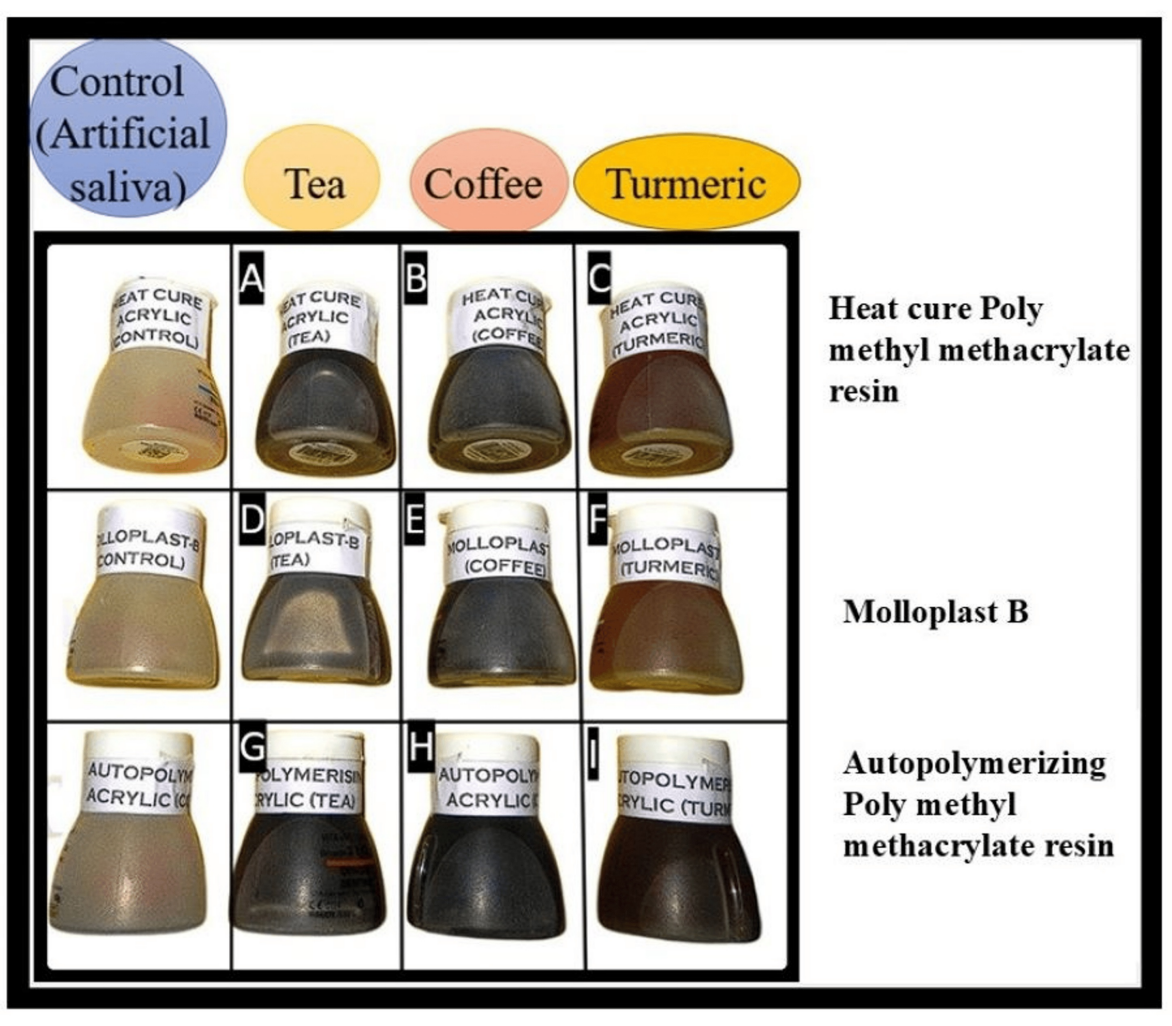
Samples immersed in different staining solutions (A) Heat-cure polymethyl methacrylate resin in tea, (B) heat-cure polymethyl methacrylate resin in coffee, (C) heat-cure polymethyl methacrylate resin in turmeric, (D) Molloplast B in tea, (E) Molloplast B in coffee, (F) Molloplast B in turmeric, (G) autopolymerizing polymethyl methacrylate resin in tea, (H) autopolymerizing polymethyl methacrylate resin in coffee, and (I) autopolymerizing polymethyl methacrylate resin in turmeric.

After the baseline measurement, the samples were divided into four subgroups, each containing six samples of each material. The samples were dipped in each staining solution and incubated at 37°C to simulate the intraoral conditions. To ensure uniform exposure of the stains, the solutions were stirred once daily. The staining solutions remain unchanged throughout the study period (Figure 3). The specimens were washed and dried using an air spray before colorimetric analysis on the 1st, 7th, and 30th days.

**Figure 4:**
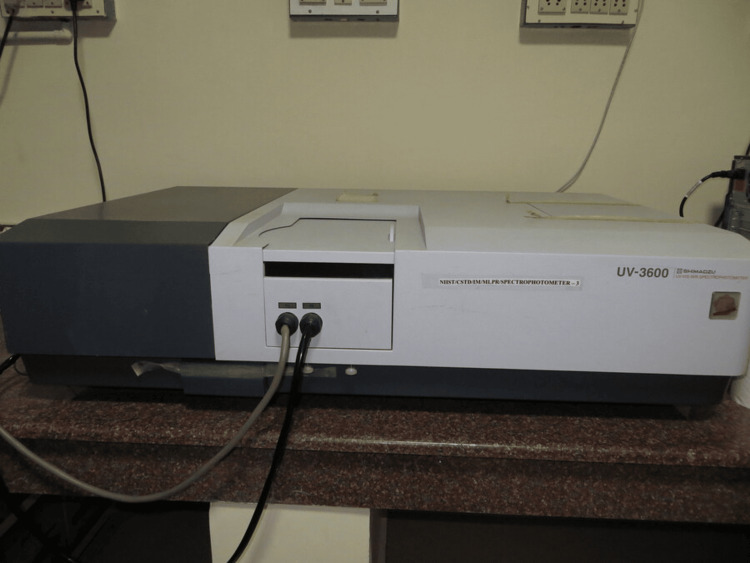
UV-3600 (Shimadzu, Kyoto, Japan) Ultraviolet-visible-near infrared (UV-VIS-NIR) spectrophotometer with an integrating sphere (BIS-603) attachment, using polytetrafluoroethylene (PTFE).

Colour measurements were done using an ultraviolet-visible-near infrared (UV-VIS-NIR) spectrophotometer (UV-3600; Shimadzu, Kyoto, Japan) with an integrating sphere (BIS-603) attachment, using polytetrafluoroethylene (PTFE) as a reference (Figure 4).

The immensity of colour difference between the two samples was calculated by the formula:

$&Delta;E^{*} = [(&Delta;L^{*})^{2} + (&Delta;a^{*})^{2} + (&Delta;b^{*})^{2}]^{1/2}$

where:

$∆L^{*} = L^{*}specimen - L^{*}standard$

$∆a^{*} = a^{*}specimen - a^{*}standard$

$∆b^{*} = b^{*}specimen - b^{*}standard$

A colour difference (ΔE) value exceeding 1 was considered perceivable by the human eye, and values below 3.7 were deemed to be clinically acceptable in the present study. Observed data were coded, tabulated, and analysed using IBM SPSS version 26 (IBM Corp., Armonk, NY) for Windows. The effect of the staining agent and the material type on the consistency of colour of denture base resins was evaluated using one-way analysis of variance (ANOVA), and significant differences between the means were analysed using Tukey's honestly significant difference (HSD) post-hoc test. The chi-square test was employed to analyse the number of samples that showed visible and clinically acceptable discolouration. The effect of time on colour stability was determined using Student’s paired t-test. A P-value less than 0.05 was considered statistically significant for all the tests.

## Results

For the heat-cure polymethylmethacrylate resin group, the colour differences in tea and coffee were statistically significant between the 7th and 30th days (P = 0.035; 0.003*) and 1st and 30th days (P = 0.013; 0.08*); and in turmeric between the 1st and 7th days (P = 0.011*), 7th and 30th days (P = 0.005*), and 1st and 30th days (P = 0.001*), respectively (Table 3).

**Table 3:** Comparison of colour stability differences among test group specimens in various staining solutions at different time intervals *The differences in mean were statistically significant (paired t-test).

Materials	Stain	Day 1 and Day 7			Day 7 and Day 30			Day 1 and Day 30		
		Mean (SD)	T-value	P-value*	Mean (SD)	T-value	P-value*	Mean (SD)	T-value	P-value*
H	T	0.494 (0.66)	1.82	0.12	2.341 (1.99)	2.87	0.035*	2.835 (1.82)	3.80	0.013*
	Co	0.278 (0.43)	1.57	0.17	1.336 (0.59)	5.51	0.003*	1.615 (0.93)	4.23	0.008*
	Tu	1.539 (0.95)	3.95	0.01*	1.724 (0.88)	4.76	0.005*	3.264 (1.26)	6.33	0.001*
A	T	1.223 (0.31)	9.66	0.00*	0.176 (0.26)	1.61	0.167	1.051 (0.07)	36.25	0.000*
	Co	0.867 (0.17)	12.16	0.00*	0.675 (0.16)	10.07	0.000*	0.193 (0.13)	3.51	0.017*
	Tu	2.137 (0.75)	6.93	0.001*	0.759 (1.23)	1.51	0.191	1.378 (0.54)	6.24	0.002*
M	T	1.827 (0.92)	4.834	0.005*	0.697 (0.88)	1.93	0.112	2.524 (0.11)	55.19	0.000*
	Co	0.618 (0.33)	4.554	0.006*	1.408 (0.43)	7.90	0.001*	0.791 (0.12)	15.50	0.000*
	Tu	0.321 (0.20)	3.853	0.012*	0.985 (0.24)	9.81	0.000*	0.664 (0.06)	24.42	0.000*

The autopolymerizing polymethylmethacrylate resin group showed statistically significant colour differences in tea between the 1st and 7th days (P < 0.001*); in coffee between the 1st and 7th days (P < 0.001*), 7th and 30th days (P < 0.001*), 1st and 30th days (P = 0.017*); and in turmeric between the 1st and 7th days (P = 0.001*) and 1st and 30th days (P = 0.002*), as shown in Table 3.

The colour differences of Molloplast B in tea were statistically significant between the 1st and 7th days (P = 0.005*) and the 1st and 30th days (P < 0.001*). In coffee and turmeric, significant differences were observed between the 1st and 7th days (P = 0.006; 0.012*), 7th and 30th days (P = 0.001; 0.000*), and 1st and 30th days, respectively (P < 0.001*), as shown in Table 3.

All discolorations obtained were perceivable to the human eye, except Molloplast B in turmeric. The mean colour shifts of all the test materials were clinically acceptable except heat-cure resin in tea (∆E = 4.142) and turmeric (∆E = 4.191) after 30 days.

## Discussion

Colour changes of dental materials may be evaluated visually or by instrumental methods. Instrumental evaluation has been reported to be more accurate in measuring slight colour changes on flat surfaces [11]. A spectrophotometric evaluation was carried out in this study to eliminate subjective errors of measurement.

CIE L*a*b* system is a uniform three-dimensional system that is commonly used for determining colour changes and is more advantageous than the Munsell colour system [12]. In this study, the colour differences were expressed in terms of CIE Lab colour coordinates. Edge losses occur for spectrophotometers when the colour of translucent materials is measured. The degree of edge loss [13] is higher when thin (less than 1.2 mm) sections are used. Hence, 2 mm thick samples were used in the present study, as denture base acrylic is a translucent material. Lee et al. [13] reported that the CIE L*, a*, and b* values were influenced by the aperture size and amount of edge loss; however, the ∆E values remain unaffected by these parameters. In the present study, the mean ∆E values for colour differences were evaluated, which also reduced the impact of edge loss effect in the results of this study.

There is evidence that intake of certain beverages, such as tea, coffee, wine, and Coca-Cola, and smoking causes staining of denture base polymers and soft liners [14]. Tea and coffee are the highest consumed beverages, and turmeric is an essential ingredient of Indian food. Hence, in this study, we used tea, coffee, and turmeric as the staining agents.

The flavonoids [1] present in tea leaves give tea its functional properties and flavour, which may be responsible for the colour changes [15]. In this study, it was observed that tea caused a significantly higher discolouration of autopolymerizing polymethylmethacrylate resin after one day, Molloplast B after seven days, and heat-cure polymethylmethacrylate resin after 30 days. This is in accordance with the studies done by Um and Ruyter [16] as well as Türker [17].

Coffee contains caffeine and caffeic acid, which are responsible for the yellowish discolouration of polymeric materials. Previous research found that less polar colourants from coffee had penetrated deeper into polymer matrices, causing increased discolouration [18]. In the present study, coffee caused considerable discolouration of heat-cured and autopolymerizing polymethylmethacrylate resin after 1 and 7 days, and Molloplast B after 30 days, similar to studies done by Oğuz ​​​​​et al. [19]. Lai et al. [20] reported that hydrophobic materials are more prone to staining by hydrophobic solutions. In the present study, coffee, a hydrophobic solution, caused a significant colour change of Molloplast B, which is a hydrophobic material.

Curcumin, a conjugated diarylheptanoid, is responsible for the orange colour and staining of turmeric solution [21]. The smaller molecular size of curcumin, coupled with the water absorption characteristics of the resin materials, has created a stronger staining effect, as discussed by Ergün et al. [12]. Um and Ruyter [16] mentioned in their study that whenever the colourant is more polar and hydrophilic, it stains denture base resins more, as denture base resins are hydrophilic. Similarly, in the present study, turmeric showed significantly higher discolouration of all three materials, and the staining increased with time.

Polymethylmethacrylate resins show the property of water sorption due to the presence of intermolecular space and the amount of residual monomer in the polymerized mass [22]. Therefore, the colour change observed here is due to the diffusion of water- and water-soluble secondary metabolites like tannins, phenols, and saponins in the interpolymeric gaps.

The colour changes of soft liners are due to alterations in their matrices, such as inhibition, hydrolysis, and decomposition of polymerizing reactions, resulting in main chain scission and branching of crosslinking [14]. Accordingly, the colour changes of soft liners, observed in the present study, should be attributed to the change of matrices of soft liners.

A survey of existing studies shows that autopolymerizing polymethylmethacrylate resin is less colour stable than heat-cure polymethylmethacrylate resin. This is attributed to the chemical composition of the monomer, change or oxidation in the amine accelerator, oxidation in the structure of the polymer matrix, and oxidation of the unreacted pendant methacrylate groups [23]. Austin et al. [24] explained that denture base materials processed by a cold polymerized method have demonstrated up to seven times higher levels of residual monomer compared to heat-polymerized materials, which is responsible for the colour changes. Purnaveja et al. [25] also showed that autopolymerized resins have colour stability inferior to that of heat-polymerized materials. Surprisingly, in the present study, although Autopolymethylmethacrylate resin showed significant but consistent colour changes over time, the colour changes remained clinically acceptable compared to heat-cured polymethylmethacrylate resin, which showed clinically unacceptable results. This needs further investigation.

The ability of the human eye to discern slight colour differences is restrained. Kuehni and Marcus [26] and Seghi et al. [27] reported that a ∆E* value equal to 1 is considered visually detectable 50% of the time, whereas a ∆E* value greater than 2 is detectable 100% of the time. Um and Ruyter [16] also suggested that the ∆E value of 1 is “visually perceptible.” Johnston and Kao [28] evaluated the appearance match by visual observation and clinical colorimetry and stated that the average colour difference (∆E) of 3.7 between compared teeth was rated as a “match” in the oral environment. Hence, in the present study, ∆E values above 1 were considered as “visible discolouration,” and ∆E values below 3.7 were considered as“clinically acceptable”.

Keskin [15] reported that there was an initial increase and then a decrease in the discolouration values of PMMA denture base polymers after immersion in coffee and tea solutions for seven days. According to him, this was due to the removal of accumulated layers. The measurements from the present study also supported this finding for autopolymerizing acrylic in tea and heat-curing polymethylmethacrylate resin in coffee.

Buyukyilmaz and Ruyter [29] reported the same level of discolouration values for seven denture base materials after 96 hours of immersion in coffee and tea solutions. Conversely, in the present study, staining of test specimens becomes more intense with time except for autopolymerizing polymethylmethacrylate resin in tea and heat-cure polymethylmethacrylate resin in coffee, and the rate of increase does not remain the same. This could probably be due to the sorption property of the resin, which gets saturated with pigments as time increases.

This study used only three staining agents; however, in clinical settings, denture discoloration is influenced by multiple factors. Also, though all samples were finely polished and visually checked for porosity, microporosities present in the samples could affect the absorption of the stains. Hence, the application of the results of the study may be interpreted with caution.

## Conclusions

Within the limitations of this study, it was concluded that significant colour shifts occurred in all three materials over time. The staining becomes more intense with time, except for autopolymerizing polymethylmethacrylate resin in tea and heat-cure polymethylmethacrylate resin in coffee. All the mean colour shifts were clinically acceptable except for heat-cure polymethylmethacrylate resin in tea after 30 days. Among the staining agents tested, turmeric affected the study materials the most. Therefore, for excellent serviceability of the prosthesis, it is essential not only for the clinician to choose the materials as per the dietary habits of the patients but also for the patients to follow proper home care maintenance.
